# Health-Related Quality of Life Among Patients With Sickle Cell Disease in an Adult Hematology Clinic in a Tertiary Hospital in Lagos, Nigeria

**DOI:** 10.7759/cureus.21377

**Published:** 2022-01-18

**Authors:** Lemchukwu Amaeshi, Olufunto O Kalejaiye, Chibuzor F Ogamba, Folasade Adelekan Popoola, Yusuf A Adelabu, Chibuikem A Ikwuegbuenyi, Ijeoma B Nwankwo, Oluwademilade Adeniran, Michael Imeh, Michael O Kehinde

**Affiliations:** 1 Internal Medicine, Montefiore Medical Center Wakefield Campus, Bronx, USA; 2 Medicine, College of Medicine, University of Lagos, Lagos, NGA; 3 Internal Medicine/Clinical Hematology, Lagos University Teaching Hospital, Lagos, NGA; 4 Internal Medicine, Lagos University Teaching Hospital, Lagos, NGA

**Keywords:** sf-36 questionnaire, lagos, bone-pain crises, health-related quality of life, sickle cell disease

## Abstract

Background

Sickle cell disease (SCD) is a genetic disease of public health concern. Improved quality healthcare has increased the life expectancy of these patients; however, they also face an increased frequency of vaso-occlusive crises and other SCD complications. These complications affect their quality of life, an area of care, which healthcare providers often overlook. We sought to determine the health-related quality of life among patients living with sickle cell disease in Lagos, Nigeria.

Materials and methods

We conducted a cross-sectional study of 198 patients with sickle cell disease who attended the adult sickle cell clinic at a tertiary hospital in Lagos, Nigeria, during the period from October 1, 2018, to February 28, 2019. A self-administered questionnaire was used to obtain the clinical and socio-demographic characteristics of the patients and the 35-item Short-Form Health Survey (SF-36) questionnaire was used to determine their health-related quality of life (HRQoL). Determinants of HRQoL were established using bivariate and multivariate regression analysis.

Results

The mean age of the 198 patients who participated in the study was 28.4±9.1 years, mean steady-state hemoglobin was 8.2 ± 1.3 g/dl, and 85 (42.9%) patients had a monthly income of 150 USD or less. In the previous year, 65 (32.1 %) and 33 (16.6%) patients, respectively, suffered one to two episodes (s) of acute bone pain crises and acute chest syndrome, and 43 (24.7%) had blood transfusion. Using the scoring system for SF-36 provided by RAND Health, role limitation due to physical health had the lowest median score of 50 (interquartile range {IQR}: 0-100). On bivariate analysis, bone pain crisis was associated with statistically significant low scores across all the 8 HRQoL domains of the SF36 questionnaire. Other variables, including having received blood transfusion, recent hospitalization, acute chest syndrome, lower level of income, and younger age, were also associated with significantly low scores. On regression analysis, bone pain crisis, level of income, and acute chest syndrome were found to be independent determinants of quality of life in the patients.

Conclusion

Sickle cell disease has a negative impact on the health-related quality of life of those affected. The presence of bone pain crisis is an important predictor of health-related quality of life in sickle cell disease patients. To improve patient outcomes, healthcare providers should take a holistic approach in evaluating and managing this disease, taking into cognizance how the complications and the financial burden of this disease impact the quality of life of affected patients.

## Introduction

Sickle cell disease (SCD) refers to a group of heterogenous hemoglobinopathies caused by a mutation of the hemoglobin gene, leading to the production of abnormal hemoglobin - hemoglobin S (Hb S). Clinical manifestations of the disease arise largely from red blood cell sickling, which leads to tissue ischemia and an increased rate of hemolysis [1,2].

Sickle cell disease is of public health concern [3]. About 300,000 babies are born with SCD each year and two-thirds of this population are found in sub-Saharan Africa (SSA) [4]. Nigeria, India, and the Democratic Republic of Congo carry half of the disease burden [4]. A retrospective study in Benin City, Nigeria, found the prevalence of SCD as 2.39% in 2012 [5]. Recently, with growing awareness of the disease and improved systems of healthcare, there has been some improvement in the survival of SCD patients well into adulthood [6,7]. While this reduces the mortality rate of SCD, these patients are nonetheless faced with debilitating SCD co-morbidities. They experience frequent vaso-occlusive crises (VOCs), resulting in frequent bone pain crises, end-organ damage to the liver, lung, spleen, kidneys, and brain, among others. These lead to frequent emergency room visits, and lengthy hospital stays in those with complications. Patients with SCD live with this burden all through their lives and it almost always influences their quality of life.

Health-related quality of life (HRQoL) is an important concept in chronic diseases, and this is defined by WHO as an assessment of a multi-dimensional concept incorporating the individual's perception of health status, psychosocial status, and other aspects of life [8]. Psychosocial and socio-economic problems resulting from the clinical burden of SCD affect QoL among individuals living with sickle cell disease [9]. This important area in the care of patients living with SCD is often ignored by healthcare providers, who focus exclusively on the clinical aspects of the disease. These patients are at a high risk of depression, suicide, drug addiction, financial troubles, and impairment in family and community social activities.

The health-related quality of life of several chronic medical conditions, including sickle cell disease, has been studied extensively [10-13]. However, only a few studies are available in the literature regarding HRQoL of adult patients living with SCD in Nigeria, the country with the highest number of adults living with the disease [10,14]. We aimed to determine the health-related quality of life (HRQoL) of adult sickle cell patients in Lagos, Nigeria

## Materials and methods

We carried out an analytical cross-sectional study designed to measure the health-related quality of life (HRQoL) of patients with sickle cell disease, who attended the adult sickle cell clinic of the Department of Internal Medicine, Lagos University Teaching Hospital Lagos, Nigeria, during the period from October 1, 2018, to February 28, 2019. Lagos is a cosmopolitan city and the commercial nerve center of Nigeria, with an estimated population of over 13 million people, of diverse ethnic and religious backgrounds [15]. Patients living with SCD who had been attending the clinic for a minimum of five years and who consented to participate in the study were consecutively recruited.

A self-administered structured questionnaire was used to collect data on the clinical and socio-demographic characteristics of the patients. This included age, gender, marital status, level of education, estimated monthly income, frequency of occurrence of vaso-occlusive crises, the presence of chronic complications of sickle cell disease, and presence of co-morbidities.

The HRQoL of the patients was determined using the Medical Outcome Study (MOS) 35-item Short-Form Health Survey (SF-36) questionnaire, developed by RAND Health, a universally accepted evaluation tool for assessing HRQoL of many chronic diseases (Appendices) [16-19]. It has 35 items to assess eight aspects of health: physical function (10 items), physical role health (four items), emotional role functions (three items), energy/fatigue (four items), emotional wellbeing (five items), social function (two items), bodily pain (two items), and general health perceptions (five items) [19]. Data collected were scored using the scoring system for SF-36, with the score of each variable ranging from 0 to 100. Higher scores indicate better quality of life and lower scores poor quality of life.

Statistical analysis

Data analysis was done using the Statistical Package for Social Sciences (SPSS) version 21 (Cary, NC: SPSS Inc.). Descriptive statistics were represented as frequencies and percentages, while normally distributed continuous variables were represented as mean and standard deviation (±SD). The median and interquartile range represented ordinal and skewed variables. We used bivariate analysis to determine the associations between socio-demographic, clinical characteristics, and the different HRQoL domains. Independent determinants of HRQoL were established using multivariate regression models. A statistically significant difference was set at a p-value <0.05.

Ethical approval for the study was obtained from the Lagos University Teaching Hospital Health Research Ethics Committee (certificate number: ADM/DCST/HREC/2534).

## Results

Socio-demographic and clinical characteristics of study participants

A total of 198 adults with sickle cell disease participated in this study. Table 1 shows the socio-demographic and clinical characteristics of the participants. The mean age of the participants was 28.4±9.1 years. About two-thirds (62.6%) of them were aged between 18 and 29 years and a half had tertiary education and were employed. Eighty-five (42.9%) of them earned less than 150 USD a month. Regarding genotype, most of the patients (89.9%) had sickle cell anemia and the mean steady-state hemoglobin was 8.2+1.3g/dl.

**Table 1:** Socio-demographic and clinical characteristics of patients with sickle cell disease Hb SS: hemoglobin SS; Hb SC: hemoglobin SC; Hb SB: hemoglobin S beta-thalassemia; AVN: avascular necrosis; SCD: sickle cell disease; n: number of participants

Characteristic		n (%)
Age	18-29	124 (62.6)
	30-39	47 (23.7)
	40-49	10 (5.1)
	50-59	10 (5.1)
	>60	7 (3.5)
	Mean ± SD (years)	28.4 ± 9.1
Sex	Male	90 (45.5)
	Female	108 (54.5)
Marital status	Single	155(78.3)
	Married	41 (20.7)
	Widowed	2 (105)
Education level	Primary	5(2.5)
	Secondary	41(20.7)
	Tertiary	107(54.0)
	Post-graduate	45(22.7)
Occupation	Employed	110 (55.5)
	Student	79(39.8)
	Unemployed	9 (4.5)
Monthly income	≤$150	85 (42.9)
	>$150-$300	33(16.7)
	>$300-$750	21 (10.6)
	>250,000	7(3.5)
	No response	52 (26.2)
Genotype	Hb SS	178 (89.9)
	Hb SC	19 (9.5)
	Hb SB thalassemia	1 (0.5)
Co-morbidities and SCD associated complication	Chronic leg ulcer	25 (12.6)
	AVN of the hip	21 (10.8)
	Hypertension	9 (4.5)
	Stroke	4 (2)
	End-stage renal disease	2 (1.0)
	Diabetes	1 (0.5)
	Seizure disorder	1 (0.5)
	Asthma	2 (1.0)
	No co-morbidities	133 (67.1)
	Hemoglobin (mean ± SD)	8.2 ± 1.3 g/dl

Sickle cell-related events/complications in the previous year

**Figure 1:**
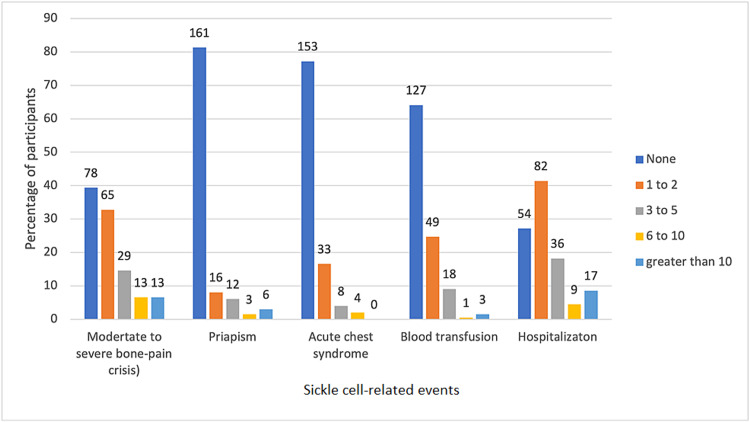
Sickle cell-related events over the previous year (n=198) n: number of participants

Moderate-to-severe bone pain crisis was the most frequent sickle cell disease-related presentation, with about 60.1% of the patients having had at least one to two episodes in the previous year. One hundred and forty-four (72.7% ) patients were hospitalized at least once and 35.8% of them had been transfused at least once in the previous year. Chronic leg ulcer was the most common sickle cell-related chronic complication, affecting about 12.6% of the population (Table 2, Figure 1).

**Table 2:** Sickle cell-related events over the previous year n: number of participants

Sickle cell-related events	n (%)				
	None	1-2	3-5	6-10	>10
Moderate-to-severe bone pain crisis	78 (39.4)	65 (32.8)	29 (14.6)	13 (6.57)	13 (6.56)
Priapism	161 (81.3)	16 (8.1)	12 (6.1)	3 (1.5)	6 (3.0)
Acute chest syndrome	153 (77.2)	33(16.6)	8 (4.0)	4 (2.0)	0 (0.0)
Blood transfusion	127 (64.1)	49 (24.7)	18 (9.1)	1 (0.5%)	3 (1.5%)
Hospitalization	54 (27.2)	82 (41.4)	36 (18.2)	9 (4.5)	17 (8.6)

Health-related quality of life of the patients

Table 3 shows the median scores and interquartile range of 198 patients in the various domains of HRQoL. The lowest score, 50 (0-100), was in role limitation due to physical health domain, the next lowest scores were energy/fatigue 60 (50-74) and pain 67.5 (42.5-90.0). The score was highest in role limitation due to emotional problem 100.0 (33.3-100.0)

**Table 3:** Health-related quality of life of sickle cell disease using the SF-36 questionnaire SF-36: 35-item Short-Form Health Survey

SF-36 domain	Median (interquartile range)
Role limitation due to emotional problems	100.0 (33.3-100.0)
Emotional well being	76.0 (64.0-88.0)
Social functioning	75.0 (50.0-875.)
General health	70.0 (55.0-80.0)
Physical functioning	70.0 (50.0-85.0)
Pain	67.5 (42.5-90.0)
Energy/fatigue	60.0 (50.0-74.0)
Role limitation due to physical health	50.0 (0.0-100.0)

Relationship between HRQoL domains and socio-demographic and clinical characteristics among study participants

The relationship between HRQoL domains, socio-demographic and clinical characteristics of the participants are shown in Table 4 and Table 5, respectively. Age and level of income had significant associations with pain, emotional well being, physical functioning, and role-limitation due to physical health (Table 4).

**Table 4:** Bivariate analysis of HRQoL domains and socio-demographics of patients RL: role limitation; HRQoL: health-related quality of life

Median score of HRQoL domains									
Variable		General health	Pain	Social functioning	Emotional well being	Energy/fatigue	Physical functioning	RL physical health	RL emotional problem
Age (years)	<30	70	57.5	75.0	76.0	60.0	75.0	50.0	100.0
	>30	70	77.5	75.0	80.0	60.0	70.0	75.0	100.0
P-value		0.784	(<0.0001)	(0.215)	(0.168)	(0.219)	(0.219)	(0.794)	(0.571)
Gender	Male	65.0	70.0	75.0	76.0	60.0	75.0	75.0	100.0
	Female	70.0	65.0	72.0	76.0	60.0	70.0	50.0	100.0
P-value		(0.98)	(0.23)	(0.78)	(0.77)	(0.95)	(0.18)	(0.05)	(0.49)
Marital status	Single	70.0	67.5	75.0	76.0	60.0	75.0	50.0	100.0
	Married	65.0	72.5	75.0	80.0	60.0	65.0	62.5	100.0
P-value		(0.39)	(0.15)	(0.35)	(0.23)	(0.95)	(0.18)	(0.80)	(0.86)
Level of education	Tertiary	70.0	67.5	62.5	76.0	60.0	70.0	50.0	100.0
	Non-tertiary	70.0	67.5	75.0	76.0	60.0	70.0	50.0	100.0
P-value		(0.55)	(0.89)	(0.326)	(0.576)	(0.55)	(0.55)	(0.682)	(0.959)
Employment status	Employed	70.0	67.5	75.0	80.0	60.0	75.0	50.0	100.0
	Unemployed	70.0	67.5	75.0	76.0	60.0	70.0	50.0	100.0
P-value		0.26	0.39	0.59	0.65	0.62	0.67	0.85	0.96
Level of income	More than $150	66.0	60.0	62.5	76.0	60.0	65.0	33.3	100.0
	$150 or less	70.0	77.5	75.0	84.0	65.0	76.0	75.0	100.0
P-value		0.43	0.05	0.06	0.03	0.08	0.02	0.01	0.35

**Table 5:** Bivariate analysis of HRQoL domains and clinical characteristics of patients RL: role limitation; HRQoL: health-related quality of life

Median score of HRQoL domains									
Variable		General health	Pain	Social functioning	Emotional well being	Energy/fatigue	Physical functioning	RL physical health	RL emotional problem
Major bone pain crisis	No	70	88.0	87.5	80.0	65.0	80.0	75.0	80.0
	Yes	65.0	55.0	62.5	72.0	55.0	65.0	25.0	72.0
P-value		0.03	<0.001	<0.001	0.02	0.007	0.001	<0.001	0.003
Priapism	No	70.0	67.5	75.0	76.0	65.0	85.0	75.0	100.0
	Yes	70.0	67.5	75.0	76.0	60.0	70.0	50.0	100.0
P-value		0.45	0.853	0.298	0.74	0.34	0.003	0.33	0.25
Blood transfusion	Yes	65.0	62.5	50.0	72.0	55.0	65.0	29.2	100.0
	No	70.0	70.0	75.0	80.0	60.0	75.0	75.0	100.0
P-value		0.152	0.04	0.008	0.02	0.462	0.01	0.06	0.125
Hospital admission	Yes	65.0	57.5	62.5	72.0	55.0	60.0	25.0	66.0
	No	70.0	77.5	62.5	76.0	60.0	70.0	50.0	100.0
P-value		0.64	0.03	0.09	0.09	0.44	0.32	0.04	0.67
Acute chest syndrome	Yes	65.0	57.5	62.5	72.0	60.0	70.0	25.0	66.7
	No	75.0	67.5	75.0	80.0	60.0	70.0	75.0	100.0
P-value		0.153	0.02	0.04	0.06	0.77	0.48	0.07	0.25
Co-morbidities/chronic complications	Yes	70.0	68.7	68.7	80.0	65.0	75.0	75.0	100.0
	No	70.0	67.5	75.0	76.0	60.0	70.0	50.0	100.0
P-value		0.77	0.83	0.80	0.25	0.67	0.70	0.85	0.30

Major bone pain crisis was significantly associated with reduced scores in all the eight domains of HRQoL of the SF-36 scale. Multivariate linear regression showed that major bone pain crisis, acute chest syndrome, and level of income were independent predictors of quality of life in patients with SCD (Table 6).

**Table 6:** Multivariate regression analysis of socio-demographic and clinical variables across the HRQoL domains Data represent standardized beta coefficients and p-value in parenthesis. RL: role limitation; HRQoL: health-related quality of life

HRQoL domains								
Independent variables	Physical functioning	RL due to physical health	RL due to emotional problem	Energy	Emotional well being	Social functioning	Pain	General health
Age	-0.179 (0.120)	-0.143 (0.233)	-0.146 (0.276)	0.06 (0.650)	0.151 (0.241)	-0.001 (0.994)	0.147 (0.237)	-0.166 (0.203)
Gender	0.097 (0.390)	-0.071 (0.545)	0.069 (0.597)	-0.081 (0.524)	0.087 (0.493)	0.059 (0.638)	-0.195 (0.113)	-0.349 (0.728)
Level of education	0.070 (0.540)	-0.864 (0.391)	0.088 (0.463)	-0.053 (0.643)	-0.015 (0.898)	0.045 (0.694)	0.069 (0.536)	0.021 (0.854)
Level of income	0.255 (0.025)	0.298 (0.013)	0.064 (0.622)	0.140 (0.267)	0.195 (0.122)	0.054 (0.668)	0.086 (0.480)	0.021 (0.854)
Bone pain crisis	0.066 (0.698)	-0.412 (0.022)	-0.291 (0.143)	-0.099 (0.606)	0.021 (0.911)	-0.057 (0.765)	-0.228 (0.219)	-0.203 (0.296)
Acute chest syndrome	-0.077 (0.491)	0.265 (0.026)	0.003 (0.980)	0.104 (0.407)	0.006 (0.960)	0.013 (0.915)	0.136 (0.261)	0.080 (0.532)
Priapism	0.195 (0.108)	0.132 (0.290)	0.259 (0.07)	0.149 (0.271)	0.104 (0.438)	0.221 (0.103)	0.029 (0.821)	0.085 (0.536)
Number of blood transfusion	-0.144 (0.274)	0..139 (0.311)	0.111 (0.465)	0.117 (0.428)	0.006 (0.967)	0.069 (0.638)	0.712 (0.479)	0.187 (0.213)
Number of hospital admissions	-0.203 (0.237)	-0.168 (0.344)	0.168 (0.396)	-0.256 (0.183)	-0.199 (0.297)	-0.298 (0.121)	-0.201 (0.278)	-0.268 (0.167)
R-square	0.389	0.308	0.106	0.145	0.153	0.150	0.204	0.122
F-test	3.551	2.413	0.914	1.342	1.422	1.395	2.024	1.099

## Discussion

We determined the health-related quality of life (HRQoL) of patients with SCD, an area of care, which healthcare givers often neglect. Those who experienced bone pain crisis had lower scores across all domains of the SF-36 scale compared to those who did not. In addition, we found the level of income, the presence of major bone pain crises, and acute chest syndrome as independent predictors of quality of life in these patients.

Sickle cell disease limited physical activity, increased fatigue, and bodily pain in our patients. Several studies on HRQoL among patients living with SCD had similar findings. A study by Dampier et al. found that pain, either acute or chronic, impairs health status and quality of life more than any other SCD-related complication [20]. Similarly, in our study, bone pain crisis was a major determinant of reduced quality of life in our patients. The score for bone pain crisis was significantly low across all eight domains. Pain is the hallmark of SCD, and it accounts for most of the emergency room visits in patients living with the disease [21]. Those living with SCD describe their pain as unimaginable, agonizing, and sometimes impossible to describe [22]. Pain in SCD, acute or chronic, reduces the quality of life in several ways and is not limited to altered mood, irritability, depression, anxiety, and sleep disturbance [23]. Work and school performance may be affected when the pain becomes significant. Unemployment and underemployment are also potential problems. This may lead to the inability to access and afford quality health care particularly in a country like Nigeria where most of her citizens pay out of pocket for healthcare. Many studies have shown that access to good healthcare services improves the quality of life of sickle cell patients by reducing the frequency of bone pain crises and its associated issues like prolonged hospitalization and frequent emergency room visits. Dampier et al. also found pain diminished SF-36 scale scores. They suggested that more effective management of persistent pain could substantially improve the quality of life for many adults with SCD.

The level of income was an independent determinant of quality of life in our study. In Lagos, a study by Okany et al. on the influence of socio-economic status on quality of life in patients living with SCD found that the frequency of bone pain crisis was significantly higher in social class III patients than in social class I and social class II (p < 0.01) [24]. In Nigeria, over 70% of its citizens have a monthly income of 150 USD or less, and over 97% do not have health insurance and have to pay out-of-pocket for healthcare. Ultimately, given the chronic nature of SCD, it becomes challenging for these patients to maintain access to and afford proper health care services [25]. Many studies have demonstrated that access to good healthcare in patients living with SCD reduces bone pain crises, which our study found to be an independent determinant of HRQoL.

Acute chest syndrome, a frequent and life-threatening complication of SCD, was an independent determinant of HRQoL in our study. The majority of patients who present at government-owned hospitals in low- and middle-income countries, such as ours come from a low socio-economic background. They prefer treating their symptoms with traditional alternatives and over-the-counter medications to presenting at the hospital because of the high cost of health care. This usually results in late disease presentation and a higher frequency of SCD complications. Acute chest syndrome is a significant cause of morbidity and mortality in patients living with SCD. Interstitial lung disease and pulmonary hypertension, which are long-term complications of ACS, may limit exercise tolerance, reduce physical functioning, and could reduce the quality of life [26]. 

We found that SCD did not have a significant negative effect on the emotional and social well-being of the patients. We expected contrary results because of the stigma attached to the disease. Some studies had similar findings [14,27]. The PISCES project, a study on HRQoL in patients living with sickle cell disease in the United States, found SCD patients had similar mental health and emotional well-being levels compared to the general population [27]. A local study by Adeyemo et al. on HRQoL of SCD among adolescents in Lagos, Nigeria, reported similar findings [14]. These studies were carried out in urban areas, where increased disease awareness in the general population through the media, health education programs, social support groups increase the knowledge and awareness of the disease, reduce fear and make them emotionally stable. Also, studies have found that patients with chronic diseases like SCD, over time, develop coping strategies towards managing it. A study by Riis et al. explained that patients with chronic diseases adapt to their disease and develop a coping mechanism, where there is a tendency to focus more on the positive experience of the disease [28]. Religion is a solid coping means and offers hope for those suffering from chronic and debilitating diseases like sickle cell disease. A study done by Anie et al. found that Nigerians with SCD had better coping strategies than those in the UK due to their socio-cultural and religious beliefs of Nigerians [29]. In Nigeria, over 95% of the populace are religious, and this may perhaps explain our findings [30].

This study is not without limitations. We conducted this research in a tertiary hospital in Lagos, a cosmopolitan city, where there is increased disease awareness among the people, with more access to education and health resources than other parts of the country. Therefore, these findings may not be representative of the country's general population. Another limitation is that we used the SF-36 questionnaire, a generic questionnaire applicable to most chronic diseases rather than a tailored, disease-specific questionnaire, in this case, SCD. Therefore, there is a need to create a validated disease-specific questionnaire to include questions unique to SCD. Lastly, given the cross-sectional study design, we could not make causal inferences from the results obtained, even those obtained from the regression analysis.

## Conclusions

This study has shown bone pain is a significant predictor of HRQoL. Therefore, developing strategies to reduce the frequency of bone pain crises will improve the HRQoL of patients living with SCD. A multi-pronged approach is therefore needed, where the patient, healthcare provider, caregiver, and the government are stakeholders in finding ways of improving the quality of life of sickle cell disease patients.

## Appendices

Medical Outcomes Study Short Form 36 Health Survey questionnaire 

This survey asks for your views about your health. This information will help keep track of how you feel and how well you are able to do your usual activities. Thank you for completing this survey! For each of the following questions, please circle the number that best describes your answer (circle one number on each line).

**Table 7:** Questionnaire - 1

1. In general, would you say your health is:	
Excellent	1
Very good	2
Good	3
Fair	4
Poor	5
2. Compared to one year ago	
Much better now than one year ago	1
Somewhat better now than one year ago	2
About the same	3
Somewhat worse now than one year ago	4
Much worse now than one year ago	5

**Table 8:** Questionnaire - 2

3. The following items are about activities you might do during a typical day. Does your health now limit you in these activities? If so, how much?			
	Yes, limited a lot (1)	Yes, limited a little (2)	No, not limited at all (3)
a. Vigorous activities, such as running, lifting heavy objects, participating in strenuous sports	1	2	3
b. Moderate activities, such as moving a table, pushing a vacuum cleaner, bowling, or playing golf	1	2	3
c. Lifting or carrying groceries	1	2	3
d. Climbing several flights of stairs	1	2	3
e. Climbing one flight of stairs	1	2	3
f. Bending, kneeling, or stooping	1	2	3
g. Walking more than a mile	1	2	3
h. Walking several blocks	1	2	3
i. Walking one block	1	2	3
j. Bathing or dressing yourself	1	2	3

**Table 9:** Questionnaire - 3

4. During the past 4 weeks, have you had any of the following problems with your work or other regular daily activities as a result of your physical health?		
	Yes, limited a lot (1)	Yes, limited a little (2)
a. Cut down the amount of time you spent on work or other activities	1	2
b. Accomplished less than you would like	1	2
c. Were limited in the kind of work or other activities	1	2
d. Had difficulty performing the work or other activities (for example, it took extra effort)	1	2

**Table 10:** Questionnaire - 4

5. During the past 4 weeks, have you had any of the following problems with your work or other regular daily activities as a result of any emotional problems (such as feeling depressed or anxious)?		
	Yes, limited a lot (1)	Yes, limited a little (2)
a. Cut down the amount of time you spent on work or other activities	1	2
b. Accomplished less than you would like	1	2
c. Didn't do work or other activities as carefully as usual	1	2

**Table 11:** Questionnaire - 5

6. During the past 4 weeks, to what extent have your physical health or emotional problems interfered with your normal social activities with family, friends, neighbors, or groups?	
Not at all	1
Slightly	2
Moderately	3
Quite a bit	4
Extremely	5

**Table 12:** Questionnaire - 6

7. How much bodily pain have you had during the past 4 weeks?	
None	1
Very mild	2
Mild	3
Moderate	4
Severe	5
Very severe	
8. During the past 4 weeks, how much did pain interfere with your normal work (including both work outside the home and housework)?	
Not at all	1
A little bit	2
Moderately	3
Quite a bit	4
Extremely	5

These questions are about how you feel and how things have been with you during the past four weeks. For each question, please give the one answer that comes closest to the way you have been feeling (circle one number on each line).

**Table 13:** Questionnaire - 7

9. How much of the time during the past 4 weeks …						
	All of the time	Most of the time	A good bit of the time	Some of the time	A little of the time	None of the time
Did you feel full of prep?	1	2	3	4	5	6
Have been a very nervous person	1	2	3	4	5	6
Have you felt calm and peaceful?	1	2	3	4	5	6
Have you felt calm and peaceful moderate?	1	2	3	4	5	6
Did you have a lot of energy?	1	2	3	4	5	6
Have you felt downhearted and blue?	1	2	3	4	5	6
Did you feel worn out?	1	2	3	4	5	6
Have you been a happy person?	1	2	3	4	5	6
Did you feel tired	1	2	3	4	5	6

**Table 14:** Questionnaire - 8

10. During the past 4 weeks, how much of the time has your physical health or emotional problems interfered with your social activities (like visiting with friends, relatives, etc.)?	
All of the time	1
Most of the time	2
Some of the time	3
A little of the time	4
None of the time	5

**Table 15:** Questionnaire - 9

11. How TRUE or FALSE is each of the following statements for you?					
	Definitely true	Mostly true	Don’t know	Mostly false	Definitely false
I seem to get sick a little easier than other people	1	2	3	4	5
I am as healthy as anybody I know	1	2	3	4	5
I expect my health to get worse	1	2	3	4	5
My health is excellent	1	2	3	4	5
